# Research on smooth path planning method based on improved ant colony algorithm optimized by Floyd algorithm

**DOI:** 10.3389/fnbot.2022.955179

**Published:** 2022-08-24

**Authors:** Lina Wang, Hejing Wang, Xin Yang, Yanfeng Gao, Xiaohong Cui, Binrui Wang

**Affiliations:** ^1^College of Mechanical and Electrical Engineering, China Jiliang University, Hangzhou, China; ^2^Key Laboratory of Intelligent Manufacturing Quality Big Data Tracing and Analysis of Zhejiang Province, China Jiliang University, Hangzhou, China

**Keywords:** Floyd algorithm, ant colony optimization, fallback strategy, multi-objective optimization, quadratic B-spline curve

## Abstract

Aiming at the problems of slow convergence and easy fall into local optimal solution of the classic ant colony algorithm in path planning, an improved ant colony algorithm is proposed. Firstly, the Floyd algorithm is introduced to generate the guiding path, and increase the pheromone content on the guiding path. Through the difference in initial pheromone, the ant colony is guided to quickly find the target node. Secondly, the fallback strategy is applied to reduce the number of ants who die due to falling into the trap to increase the probability of ants finding the target node. Thirdly, the gravity concept in the artificial potential field method and the concept of distance from the optional node to the target node are introduced to improve the heuristic function to make up for the fallback strategy on the convergence speed of the algorithm. Fourthly, a multi-objective optimization function is proposed, which comprehensively considers the three indexes of path length, security, and energy consumption and combines the dynamic optimization idea to optimize the pheromone update method, to avoid the algorithm falling into the local optimal solution and improve the comprehensive quality of the path. Finally, according to the connectivity principle and quadratic B-spline curve optimization method, the path nodes are optimized to shorten the path length effectively.

## Introduction

The path planning of mobile robot is to plan the optimal path from the starting point to the target point in the specified area (Chen et al., 2020). At present, path planning algorithms is mainly presented in the form of traditional algorithms and intelligent algorithms. The traditional algorithms include the A^*^ Algorithm (Xiong et al., 2020), Tabu Search (TS) (Khaksar et al., 2012), and D^*^ Algorithm (Yao et al., 2021), etc. The intelligent algorithms include Ant Colony Optimization (ACO) (Wang, 2020), Particle Swarm Optimization (PSO) (Wang et al., 2020a), Genetic Algorithm (GA) (Chen and Gao, 2020), etc.

Intelligent algorithms can also be subdivided. Among them, the ant colony algorithm and particle swarm optimization algorithm belong to the swarm intelligent algorithm. Swarm intelligent algorithm has been a hot spot in path planning. There are two modes of swarm intelligence, namely, ant colony algorithm and particle swarm optimization algorithm. Swarm intelligence mainly refers to the intelligent behavior of many non-intelligent individuals in a group through simple cooperation. Swarm intelligence is applied to path planning, taking the ant colony algorithm as an example. It shows that a single ant in the ant colony has no intelligence, but through cooperation to form a complete system, it evolves into an intelligent whole that can explore the optimal path in a complex environment. Therefore, it is widely studied and applied in path planning.

Swarm intelligence is mainly manifested in five principles: (1) Proximity principle; (2) Quality principle; (3) The principle of diverse response; (4) Stability principle; (5) Adaptability principle.

Swarm intelligence also has four features.

(1) The control of swarm intelligence is decentralized, and there is no unified control center, so it can adapt to various environments and has strong robustness. For example, the ant colony algorithm can carry out path planning in various complex environments and obtain the optimal path.
(2) Each individual in the swarm can communicate by changing the environment, which has good scalability. For example, the ants change the pheromone content in the environment by leaving pheromones on the path, to realize communication with other individuals.
(3) The behavior of individuals in the swarm or the rules they follow are very concise, so it is very convenient to realize swarm intelligence. For example, individuals in the ant colony only need to follow the state transition rules to find the path and leave pheromones to inform the latecomers.
(4) The complex behavior of a swarm is the result of individual communication and cooperation. Under the guidance of appropriate rules, swarm intelligence can play a role in some form of emergence through communication and cooperation. For example, individuals in the ant colony interact through pheromones and then complete path exploration. Then pheromone update mechanism plays a role in guiding the ant colony to optimize the path further and finally get the optimal path.

Ant colony algorithm in swarm intelligence fully reflects the characteristics of swarm intelligence. It is simple to set parameters, suitable for various complex environments, and has strong robustness. Therefore, it is widely used in robot path planning. In this paper, the ant colony algorithm will be deeply studied and optimized.

Italian scholar Marco Dorigo proposed the ant colony algorithm in 1992. The algorithm was derived from the path finding behavior of ants looking for food sources in nature (Mac et al., 2016). The most prominent feature of the ant colony algorithm is the positive feedback mechanism (Zhang et al., 2021) which is conducive to obtaining the optimal solution quickly. Then, the ant colony can change the environment by releasing pheromone, so as to communicate indirectly (Yi et al., 2019). At last, the ant colony adopts the distributed computing method to search the path (Zheng et al., 2020), and the parallel computing is carried out by multiple individuals at the same time. Nevertheless, the defects of slow convergence speed and easy to fall into the local optimal solution cannot be ignored (Yang et al., 2019).

For the defects of the ant colony algorithm, many researchers have proposed optimization schemes that can be divided into three categories. (1) In consideration of the slow convergence speed of the ant colony algorithm, improve the initial pheromone allocation method, or improve the state transition probability matrix, such as Luo et al. (2020) and Li et al. (2021); etc. (2) In order to optimize the defect of the ant colony algorithm that it is easy to fall into local optimal solution, the pheromone matrix updating method is optimized or pheromone concentration is limited, such as Akka and Khaber (2018) and Wang et al. (2020), etc. (3) Many schemes to improve the path smoothness of ant colony algorithm have been proposed. There are mainly two ways: improving the heuristic function and optimizing the path nodes, such as Dai et al. (2019) and Yang et al. (2019), etc. Some optimization schemes will be introduced in detail below.

To improve the ant colony algorithm, there are a lot of optimization schemes (Akka and Khaber, 2018; Luo et al., 2020; Li et al., 2021). In Luo et al. (2020), an improved ant colony algorithm was proposed. The algorithm constructs unequally distributed initial pheromone in the early stage of path planning. At the same time, the pseudo-random state transition rule is used to select the trail. The deficiency is that the algorithm only sets the initial pheromone according to the position information of the node, which is not conducive to avoiding obstacles in the process of the ant search path, and the guidance of the ant colony is not direct enough. In Li et al. (2021), an improved algorithm based on turning angle constraint was proposed. Firstly, the initial pheromone concentration between the starting node and the target node is increased. Then, the evaluation function and rotation constraint factor of the A ^*^ algorithm is added to the heuristic function. The nodes with the optimal path length and rotation number can be selected in the next step. Finally, in the pheromone updating part, the distribution principle of the wolf swarm algorithm is introduced to strengthen the influence of a high-quality population. The algorithm proposed by Li effectively avoids falling into optimal local solutions, but the convergence speed in a complex environment cannot meet the requirements. In Akka and Khaber (2018), an improved ant colony optimization algorithm was proposed. The algorithm uses stimulus probability to help ants select the following grid, and uses new heuristic information to improve visibility accuracy. In addition, the improved algorithm adopts new pheromone updating rules and dynamically adjusts the evaporation rate, which accelerates the convergence speed and expands the search space. This algorithm does not consider the requirements of path smoothness when effectively accelerating the convergence speed, which is not conducive to reducing the energy consumption and mechanical loss of the robot.

In summary, to solve the problems of slow convergence rate and easily fall into the local optimal solution of ant colony algorithm, this paper proposes an improved algorithm.

(1) For the difficulties in Luo et al. (2020), the Floyd algorithm is introduced to generate the guidance path. The path is a feasible path without collision with obstacles. Setting the initial pheromone based on the track can help the ant colony avoid blind search and take into account the obstacle avoidance needs.
(2) Considering that the ants easily fall into the deadlock and self-locking state, the fallback strategy is proposed to reduce the number of dead ants and help improve the success rate of the algorithm to solve the way.
(3) For the problems that have not been solved in Li et al. (2021), the APF method and the concept of the distance between the optional node and the target node are introduced to optimize the structure of the heuristic function, which improves the state transition probability and accelerates the convergence rate.
(4) Given the shortcomings of Akka and Khaber (2018), the connectivity principle and quadratic B-spline curve optimization method are proposed to optimize the corner nodes, further shortening the path length and reducing the mechanical loss of the robot in the working process.
(5) Moreover, this paper proposes a multi-objective optimization method, taking into account the path length, path safety, and path energy consumption, to solve the bearing with the highest comprehensive quality. The pheromone updating method is improved based on the multi-objective optimization method and dynamic principle, which prevents the algorithm from falling into the local optimal solution to the greatest extent.

The rest of this paper is as follows. The second part briefly describes the two-dimensional grid environment modeling method, which is a crucial environment for algorithm operation. The third part introduces the core part of the classic ant colony algorithm. The fourth part gives the progress measures of the algorithm in detail. In the fifth part, the classic ant colony algorithm and the improved algorithm are compared and analyzed. The sixth part summarizes the contributions and shortcomings of the improved algorithm, and briefly looks forward to future work.

## Environment modeling

**Figure 1:**
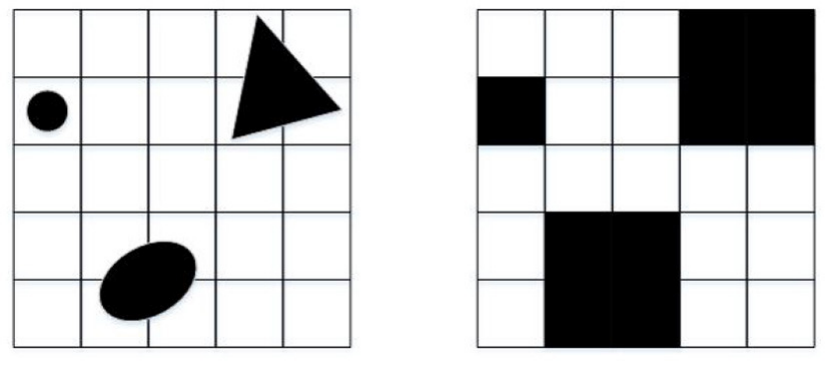
Obstacle rule processing.

Environment modeling is the basic part of a path planning algorithm (Mac et al., 2016). The grid method is used in mobile robot path planning algorithms because of its simple modeling method, easy programming, and ability to express irregular obstacles. It is a commonly used environmental modeling method (Ouyang and Yang, 2014). The grid method converts environmental information into grid form (Zhang et al., 2019), and distinctive blocks are regularly processed and properly expanded, as shown in Figure 1, which greatly reduces the difficulty of path planning.

In the grid map, the white grid is the free space and optional node, represented by “0.” The black grid is the obstacle space and belongs to the tabu node, represented by “1.”

In addition, the selection of grid size is also a key factor of the algorithm. If the grid is too small, the map resolution is high, which is not conducive to fast decision-making. If the grid is too large, the map resolution will be low, which is conducive to quick decision-making. Still, it cannot guarantee a viable path in the dense obstacle environment.

Although the grid sequence number method saves more memory, it is not conducive to the rapid iteration of the ant colony algorithm (Xiao et al., 2021). To ensure the convergence speed, the grid sequence number will be converted to coordinate (*x, y*), and the conversion formula is as follows.


(1) $$\left\{ \begin{array}{l} x=\;\mathrm{mod}\;(i,M)-0.5\\ y=M-ceil(i/M)+0.5 \end{array}\right.$$

In the formula, *M*is the map size, mod is the solution function that returns the abscissa of the grid, and the*ceil*process returns the grid ordinate (Ali et al., 2020).

**Figure 2:**
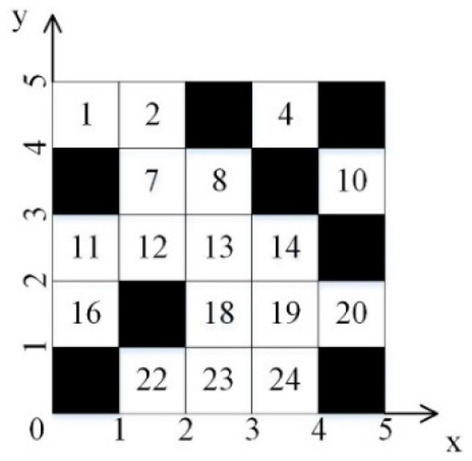
Grid coordinate diagram.

The coordinate form of the grid map is shown in Figure 2.

## Ant colony optimization algorithm

Ant colony algorithm is derived from the path finding behavior of ants looking for food sources in nature, which has strong robustness in complex environment. The classic ant colony algorithm is easy to implement, the parameter setting is convenient, and the requirement for the computing environment is low (Zhang et al., 2020). The two important mechanisms of the ant colony algorithm are positive feedback and pheromone communication. The positive feedback mechanism guarantees the convergence of the ant colony algorithm. The higher the path quality, the more pheromones will accumulate when the pheromone is updated, which will encourage more ants to choose and realize the fast convergence of the algorithm. The pheromone communication mechanism is indirect communication for ant colony individuals. Ants leave pheromones on the path they traversed. Other individuals combine known environmental information and pheromones on the course to become new prior knowledge, which will help ant colonies reduce blind search and find target nodes faster.

These two mechanisms evolve into two key links in algorithm implementation: state transition probability (Chen et al., 2021) and pheromone update (Li and Wang, 2020).

### State transition probability

Ants need to go through many intermediate nodes in the process of finding the path. For the selection of each intermediate node, the state transition probability matrix of the optional node should be established first, and then select from the probability matrix by roulette operation (Wang et al., 2020b).

The state transition probability is shown in equation (2).


(2) $$\begin{array}{ccc} P_{ij}^{k}(t) & = & \{\begin{array}{cc} \frac{\tau_{ij}^{\alpha }(t)\eta_{ij}^{\beta }(t)}{\underset{j\in allowed_{k}}{\sum^{\text{​}}}\tau_{ij}^{\alpha }(t)\eta_{ij}^{\beta }(t)} & s\in allowed_{k}\\ 0 & s\notin allowed_{k} \end{array} \end{array}$$

(3) $$\begin{array}{rcl} \eta_{ij} & = & \frac{1}{d_{ij}} \end{array}$$

(4) $$\begin{array}{rcl} d_{ij} & = & \sqrt{{\left(x_{i}-x_{j}\right)}^{2}+{\left(y_{i}-y_{j}\right)}^{2}} \end{array}$$

Where, τ_*ij*_ is the pheromone content from node *i* to node *j*, η_*ij*_ is the heuristic function, *d*_*ij*_ is the Euclidean distance from node *i* to node *j*, α is the pheromone heuristic factor, β is the expected heuristic factor, and *allowed*_*k*_ is the set of optional nodes in the next step (Xiong et al., 2021).

### Pheromone update mode

Individuals in the ant colony will leave pheromones when passing through each path. As a prior knowledge of subsequent individuals, ants communicate indirectly through the pheromones. After several iterations, the ants traverse the map, and the pheromone content of the path indicates the quality of the trajectory. The higher the quality of the path pheromone concentration is higher. In the algorithm implementation process, to facilitate calculation, a pheromone update is placed after each iteration of the ant complete path search.

The pheromone update method is shown in equation (5).


(5) $$\begin{array}{rcl} \tau_{ij}\left(t+1\right) & = & \left(1-\rho \right)*\tau_{ij}\left(t\right)+\Delta \tau_{ij}\left(t\right) \end{array}$$

(6) $$\begin{array}{rcl} \Delta \tau_{ij}\left(t\right) & = & \overset{m}{\underset{k=1}{\sum }}\Delta \tau_{ij}^{k}\left(t\right) \end{array}$$

(7) $$\begin{array}{ccc} \Delta \tau_{ij}^{k}(t) & = & \{\begin{array}{cc} \frac{Q}{L_{k}} & tour(i,j)\in tour_{k}\\ 0 & tour(i,j)\notin tour_{k} \end{array} \end{array}$$

Where, ρ is the pheromone volatilization rate, Δτ_*ij*_(*t*) is the total pheromone increment of the path in this iteration, $\Delta \tau_{ij}^{k}\left(t\right)$ is the pheromone increment brought by the *k*-th ant, *m* is the number of ants, *Q* is the pheromone increase intensity, and *L*_*k*_ is the path length traveled by the *k*-th ant (Tao et al., 2021).

## Improvement of ant colony optimization algorithm

### Initial pheromone matrix

The Floyd algorithm is named after Robert Floyd (Hao and He, 2008), one of the founders. Floyd algorithm is a dynamic programming algorithm, suitable for dense maps, simple and effective, and easy to implement. Its efficiency is higher than the Dijkstra algorithm (Shi and Wang, 2009). Taking the optimal path obtained by the Floyd algorithm as the guiding path of the ant colony algorithm can help set the initial pheromone matrix with a guiding effect (Tian, 2021a).

Floyd algorithm can calculate the shortest path between each node in the map environment, and the core idea is to solve the shortest path matrix (Lyu et al., 2021). There are only two possible shortest paths from node*i*to node*j*. One is the Euclidean distance of two nodes. That is, two nodes are connected, and the other is from node *i* to node *j* through several intermediate nodes (Yang, 2020). Therefore, *Dis*(*i, j*) is set as the Euclidean distance from node*i*to node*j*, and then all nodes*k*except these two nodes are judged. If*Dis*(*i, k*)+*Dis*(*k, j*) < *Dis*(*i, j*)holds, it is proved that the path from node*i*to node*k*and then to node *j*is shorter than the path from node*i*to node*j*, then let *Dis*(*i, j*) = *Dis*(*i, k*)+*Dis*(*k, j*). After traversing node *k*, the shortest distance from node *i* to node *j* is recorded in*Dis*(*i, j*).

The implementation of the Floyd algorithm is as follows.

(1) Initialize the shortest distance matrix*Dist*as the adjacency matrix of the map, and the path node matrix *path*is empty. The elements in the adjacency matrix are initialized to infinity. If two nodes have edges, the corresponding elements in the matrix are set as weight values.
(2) For node *i* to node *j*, traversing the remaining nodes to determine whether there is node*k*makes the distance from node *i* to node *k* and then to node *j* shorter than the known path. If it exists, update matrix*Dist*and matrix*path*.

The state transition equation is shown in equation (8).


(8) $$\begin{array}{l} Dist(i,j)\\ =\{\begin{array}{cc} Dist(i,j) & Dist(i,k)+Dist(k,j)\geq Dist(i,j)\\ Dist(i,k)+Dist(k,j) & Dist(i,k)+Dist(k,j)<Dist(i,j) \end{array} \end{array}$$

After determining the starting node and the target node, the Floyd algorithm can quickly obtain the optimal path. Then take the generated path as the guidance to change the pheromone content on the path so that it is different from other paths. Because the ant will be affected by pheromone when choosing the path, it is easier to choose the guidance path. The pheromone difference between the guide path and other paths will make the ant tend to the former to quickly find the target node. The initial pheromone matrix is set as follows.


(9) $$\begin{array}{c} \tau_{ij}(0)=\{\begin{array}{cc} k*C & tour(i,j)\in tour_{F}\\ C & tour(i,j)\notin tour_{F} \end{array} \end{array}$$

Where, τ_*ij*_(0)is the initial pheromone matrix. *tour*_*F*_is the guiding path generated by the Floyd algorithm, and the pheromone concentration of the guiding path is set to*k*times of other paths.

The APF has also been used to generate the guidance path of the ant colony algorithm. Therefore, under the same conditions, the path planning results of APF method and Floyd algorithm are compared. The results are shown below.

**Figure 3:**
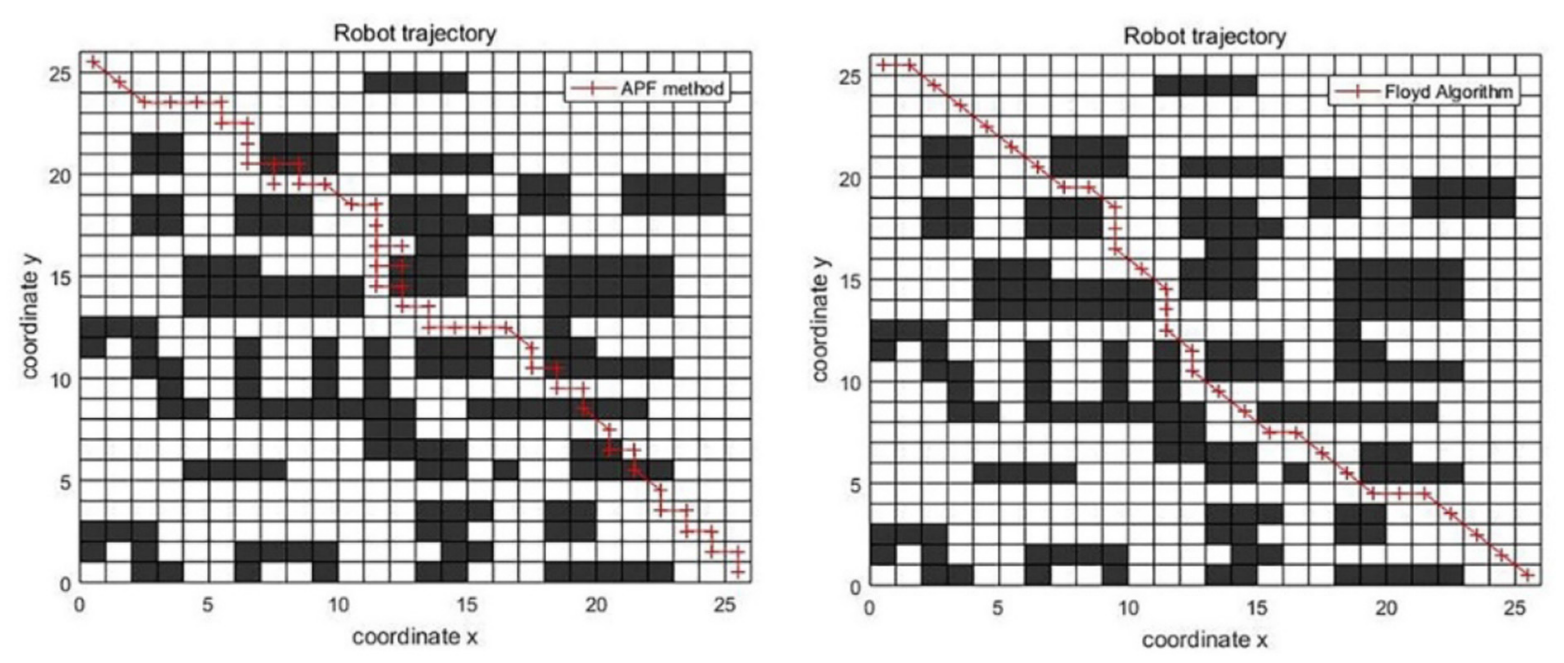
Comparison of boot paths.

According to Figure 3 and Table 1, the path of APF method will pass through obstacles, which is not allowed, while the path of Floyd algorithm fully realizes the requirements of obstacle avoidance. In addition, the Floyd algorithm has few redundant nodes, and the length is only 51.40 % of the APF method. The Floyd algorithm is much better than the APF method. Therefore, introducing the optimal path of the Floyd algorithm as the guiding path will help the ant colony algorithm quickly find the target node and accelerate the convergence speed of the algorithm.

**Table 1:** Comparison of boot paths length.

	**APF method**	**Floyd algorithm**
Optimal path length	74.4853	38.2843

### Ant fallback strategy

The ants often encounter deadlock problems when exploring paths (Dai et al., 2019), including self-locking and deadlock caused by obstacles. The deadlock problem will cause excessive death of ants, weaken the ability of the ant colony to explore the path, and slow down the convergence speed of the algorithm (Tian, 2020b; Wang, 2020).

**Figure 4:**
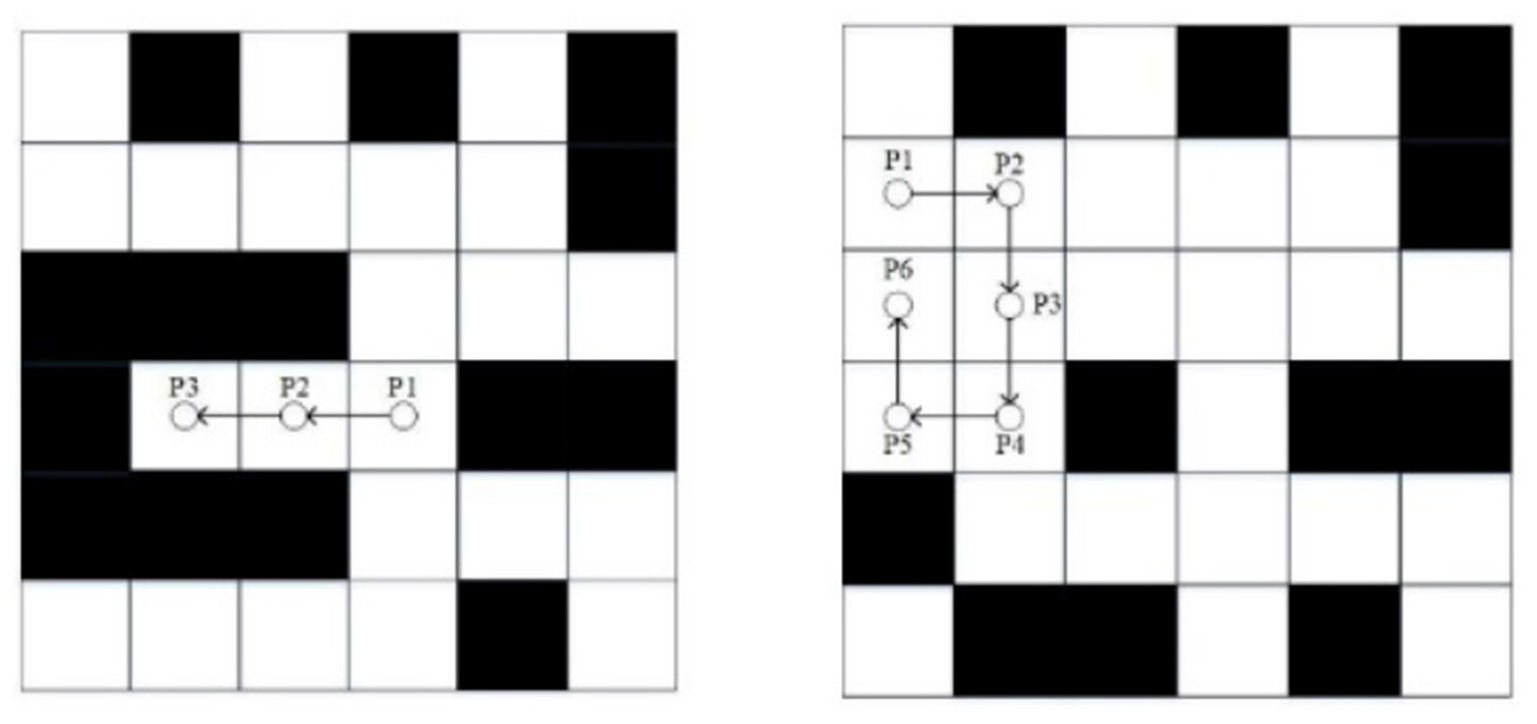
Deadlock and self-locking.

Obstacles that will form ant deadlocks are usually concave. Because the ant follows the rule of putting the passed nodes in the tabu list when exploring the path to reduce the generation of redundant nodes, when ants encounter concave obstacles, this rule will make ants unable to stay away from the obstacles and thus trapped near the obstacles. Self-locking is due to that ants have no clear direction of the target node at the beginning of the iteration, only blind search, and ultimately face the plight of no optional nodes. The above two deadlock problems are shown in Figure 4.

The particles in Figure 4 are the ants searching path. On the left side of Figure 4, the ant at node P1 chooses the left node P2. It cannot retreat away from the obstacles because of the tabu list rules. The ant can only continue to select the left node P3, and finally, it is trapped in the barrier. On the right side of Figure 4, the ant at node P1 does not get a clear direction of the target node and can only choose the next node based on roulette. The ant follows the series of nodes like*P*1 → *P*2 → *P*3 → *P*4 → *P*5 → *P*6and finally, the ant is trapped in a self-locking dilemma. In the classic ant colony algorithm, ants are usually discarded after they fall into the deadlock dilemma so that subsequent ants continue to search the path. The situation when ants fall into deadlock can be described by the following formula.


(10) $$\begin{array}{r} allowed_{i}\cap Obs=allowed_{i} \end{array}$$

Where, *allowed*_*i*_is the list of optional nodes, *Obs*is the tabu list.

**Figure 5:**
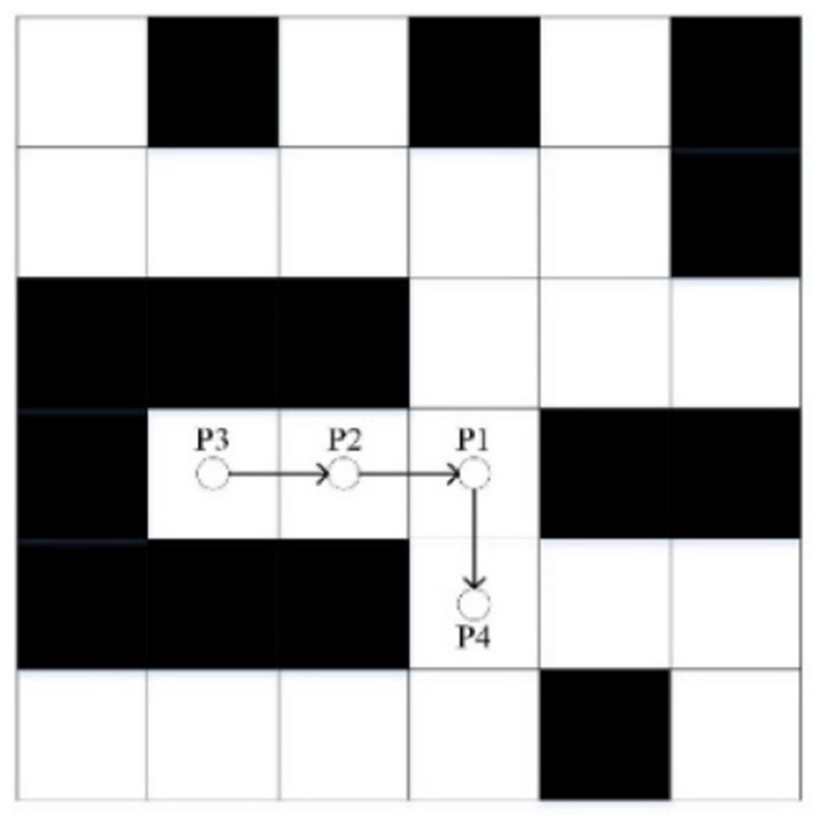
Fallback strategy.

To solve the deadlock problem, the ant fallback strategy is proposed. When the ant has no optional node and has not reached the target point, the fallback strategy is implemented. That is, the current node is added to the tabu list and returned to the previous node, and the pheromone concentration at the current node is reduced. If there are new optional nodes at this time, the fallback strategy will end. If not, continue to execute the fallback strategy until there are optional nodes for ants to select. The fallback strategy is shown in Figure 5.

In Figure 5, the ant at node P3 falls into the deadlock and starts to perform the fallback strategy. The node P3 is added to the tabu list, and the ant returns to node P2. There is no optional node for the ant to choose, so the ant continues to implement the strategy. When returning to node P1, the ants find new optional nodes. At this time, it ends the execution of the fallback strategy. The ant selects the node P4 by the roulette rule and continues to explore new paths. Since the trap has been added to the tabu list, subsequent ants will no longer fall into the deadlock dilemma here. The treatment of the self-locking dilemma is similar (Tian and Chen, 2021a).

The pheromone update method when executing the fallback strategy is as follows.


(11) $$\begin{array}{r} \tau_{ij}\left(t+1\right)=\left(1-\lambda \right)*\tau_{ij}\left(t\right) \end{array}$$

Where, λ is the pheromone penalty evaporation coefficient, which reduces the pheromone concentration of the trap nodes and helps the ant avoid the trap.

### Heuristic function optimization strategy

To better solve the slow convergence problem of the ant colony algorithm, some optimization schemes are proposed for the heuristic function.

The heuristic function of the ant colony algorithm is the reciprocal of the Euclidean distance between the current node and the optional node, as follows.


(12) $$\begin{array}{r} \eta_{ij}=\frac{1}{d_{ij}},d_{ij}=\sqrt{{\left(x_{i}-x_{j}\right)}^{2}+{\left(y_{i}-y_{j}\right)}^{2}} \end{array}$$

The heuristic function does not contain the information of the target node, and the ant lacks guidance in finding the path, which is easy to search blindly, resulting in the slow convergence of the algorithm. This paper proposes the concept of distance between the optional node *j* and target node *E*, replacing the original heuristic function, as follows.


(13) $$\begin{array}{r} \eta_{ij}=\frac{1}{d_{jE}},d_{jE}=\sqrt{{\left(x_{E}-x_{j}\right)}^{2}+{\left(y_{E}-y_{j}\right)}^{2}} \end{array}$$

The heuristic value of the optional node closest to the target node is the largest, and the probability of being selected is also the largest. With the information of the target node, the ant colony has a clear direction in exploring the path, and the convergence speed will be accelerated.

Based on the new heuristic function, considering further improving the convergence speed of the algorithm, the APF method is an option. The APF method has the advantages of low calculation and fast convergence speed, so it is considered to optimize the heuristic function (Wang et al., 2020c).

**Figure 6:**
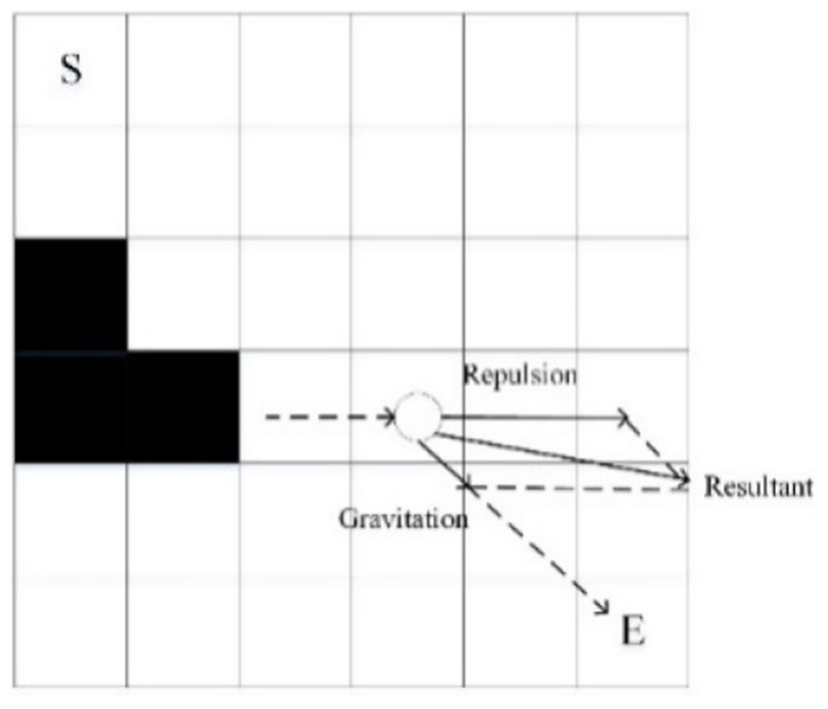
Artificial potential field method.

As one of the widely used path planning algorithms, the APF method was first proposed by Khatib. O in 1985 (Pan et al., 2019). The main idea of the APF method is to regard the motion environment of the robot as a virtual force field (Li and Wang, 2022). The target node and obstacles generate gravitational and repulsive forces, respectively, in the robot, and the motion of the robot is controlled by the resultant force. The effect of the APF method is shown in Figure 6. The particle is a mobile robot (Wang and Wang, 2020a).

After simulation experiments, the gravity concept of the target node to the robot in the APF method is used to optimize the heuristic function, which is as follows.


(14) $$\begin{array}{r} Gra=sigma*\sqrt{{\left(x_{E}-x_{j}\right)}^{2}+{\left(y_{E}-y_{j}\right)}^{2}} \end{array}$$

(15) $$\begin{array}{r} d_{jE}=1/\sqrt{{\left(x_{E}-x_{j}\right)}^{2}+{\left(y_{E}-y_{j}\right)}^{2}} \end{array}$$

(16) $$\begin{array}{r} \eta_{ij}=\frac{power\left(0.5,Gra\right)}{d_{jE}} \end{array}$$

Where, *Gra*is the gravitational effect of the target node on the optional node, *sigma*is the gravitational constant, *power*is the power function that returns the value of the exponential power of the given bottom number. *j* is the optional node and *E* is the target node.

In the early iterations, the pheromone is accumulating, and the state transition probability is dominated by the heuristic function. In the middle and late iterations, the ants complete the path exploration, and the pheromone accumulates a lot. The pheromone dominates the state transition probability. Therefore, the heuristic function needs to be adjusted adaptively to match the pheromone matrix, so as to improve the update mode of the state transition probability matrix.

The adaptive adjustment of the heuristic function is related to the number of iterations. Therefore, the normal distribution function is introduced and combined with the heuristic function, as shown below.


(17) Nd_function=e(−((k/K) ^2)/2)

(18) $$\begin{array}{c} P_{ij}^{k}(t)=\{\begin{array}{cc} \frac{\tau_{ij}^{\alpha }(t)*{\left(Nd\_function*\eta_{ij}(t)\right)}^{\beta }}{\underset{j\in allowed_{k}}{\sum^{\text{​}}}\tau_{ij}^{\alpha }(t)*{\left(Nd\_function*\eta_{ij}(t)\right)}^{\beta }} & s\in allowed_{k}\\ 0 & s\notin allowed_{k} \end{array} \end{array}$$

Where, *Nd*_*function*is the deformation of the standard normal distribution function, omitting the coefficient, *k*is the current number of iterations, *K*is the maximum number of iterations, $P_{ij}^{k}\left(t\right)$is the optimized state transition probability.

### Optimization strategy of pheromone updating method

In the classic ant colony algorithm, the pheromone updating method is only related to the path length. The shorter the path, the higher the pheromone increment. The updating method ignores other requirements, such as path security and energy consumption. In addition, the pheromone volatilization coefficient is constant and does not dynamically update with iterations. In the late iterations, the optimal path has been fixed. The behavior of finding a better path has stopped, which causes the local optimal solution (Wang and Wang, 2020b).

To solve the above problems, this paper proposes a pheromone updating method based on the multi-objective optimization method (Guo et al., 2020) and dynamic principle (Tian et al., 2020; Tian, 2021b).

Multi-objective optimization has been used in other path planning algorithms, mainly to improve the quality of the algorithm. Based on the idea of multi-objective optimization, this section puts forward three optimization objectives: path length, path security, and path energy consumption, which are used as the standard to update the pheromone matrix. Where, the path length is the sum of the distances of the path nodes, denoted as *Length*; path safety is the number of dangerous nodes on the path, denoted as *Risk*; path energy consumption depends on the number of turns and turning angles of the path, denoted as*Consumption*. The multi-objective optimization function is shown below.


(19) $$\begin{array}{rcl} Length & = & \overset{j=E}{\underset{i=S}{\sum }}d_{ij} \end{array}$$

(20) $$\begin{array}{rcl} Risk & = & \sum D\text{\_}nodes \end{array}$$

(21) $$\begin{array}{rcl} Consumption & = & \sum 0.5*N\text{\_}corner+0.5*T\text{\_}angle \end{array}$$

(22) $$\begin{array}{l} \begin{array}{ccc} J\_quality & = & k_{1}*Length+k_{2}*Risk+k_{3} \end{array}\\ \;\begin{array}{cc} {}* & Consumption \end{array} \end{array}$$

(23) $$\begin{array}{rcl} k_{1}+k_{2}+k_{3} & = & 1 \end{array}$$

Where, *Length* is the sum of distances of all nodes in the path. *Risk*is the sum of dangerous nodes that the path passes. Dangerous nodes refer to nodes whose ratio of optional nodes to obstacle nodes is <1. *Consumption*is the sum of the number of corners and the turning angles of the path. *J*_*quality*is the comprehensive quality, a comprehensive index composed of path length, path safety, and path energy consumption with different proportions. The smaller the value is, the higher the comprehensive quality of the path is.

Replacing the path length with the comprehensive quality is the way to realize the multi-objective optimization idea. The improved pheromone update method is as follows.


(24) $$\begin{array}{c} \Delta \tau_{ij}^{k}(t)=\{\begin{array}{cc} \frac{Q}{J_{best}}+\frac{b*Q}{BEST} & tour(i,j)\in tour_{best}\\ \frac{Q}{J_{worst}}-\frac{w*Q}{WORST} & tour(i,j)\in tour_{worst}\\ \frac{Q}{J} & tour(i,j)\in tour_{other} \end{array} \end{array}$$

(25) $$\begin{array}{r} \Delta \tau_{ij}\left(t\right)=\overset{m}{\underset{k=1}{\sum }}\Delta \tau_{ij}^{k}\left(t\right) \end{array}$$

(26) $$\begin{array}{r} \tau_{ij}\left(t+1\right)=\left(1-\rho \right)*\tau_{ij}\left(t\right)+\Delta \tau_{ij}\left(t\right) \end{array}$$

Where, *J*_*best*_ is the comprehensive quality of the local optimal path, *b*is the number of ants on the local optimal path, *BEST*is the comprehensive quality of the global optimal path. *J*_*worst*_is the comprehensive quality of the local worst path, *w*is the number of ants on the local worst path, *WORST*is the comprehensive quality of the global worst path. The optimal path, the worst path and other paths update the pheromone according to the three forms in equation (24), respectively.

The optimized pheromone updating method is based on the comprehensive quality of the path. The reward and punishment system is implemented for the optimal path and the worst path, and the pheromone gap between them gradually increases. The subsequent ants will be more inclined to the optimal path, which helps to accelerate the convergence speed of the algorithm (Dai et al., 2022).

In the late iteration of classic ant colony algorithm, the pheromone accumulation is completed, and ants are affected by pheromone, so it is difficult to continue to explore other paths, and the local optimal solution needs to be solved (Tian and Chen, 2021b).

In this paper, the concept of dynamic updating volatility coefficient is proposed. In the late iterations, if the quality of the optimal path of five consecutive iterations does not change, the volatility coefficient is dynamically updated, which can increase the volatilization of pheromone and weaken the attraction of pheromone. This helps ants explore other paths. The dynamic volatility coefficient is shown below.


(27) $$\begin{array}{c} \rho =\{\begin{array}{cc} 1.2*\rho & J(k)=J(k-5),k\geq 35\\ 0.8 & \rho \geq 0.8 \end{array} \end{array}$$

Where, *J*(*k*)and*J*(*k*−5)are the comprehensive quality of the optimal path of this iteration and five iterations ago, respectively. In the late iterations, if the *J*_*best*_ does not change in the five consecutive iterations, the volatilization coefficientρ increases, and the volatilization is enhanced, which makes the ants explore better solutions. The upper limit of the volatilization coefficient is 0.8.

### Path smoothing

The path obtained by classic ant colony algorithm has many redundant nodes and corners, which not only affects the path length, but also is not conducive to reducing the energy consumption of the robot. Therefore, the improved ant colony algorithm needs to optimize the path nodes. In this paper, the connectivity processing and quadratic B-spline curve optimization method are proposed to optimize the nodes, which further shortens the path length and reduces the energy consumption of the robot (Tian, 2020a; Tian et al., 2021).

**Figure 7:**
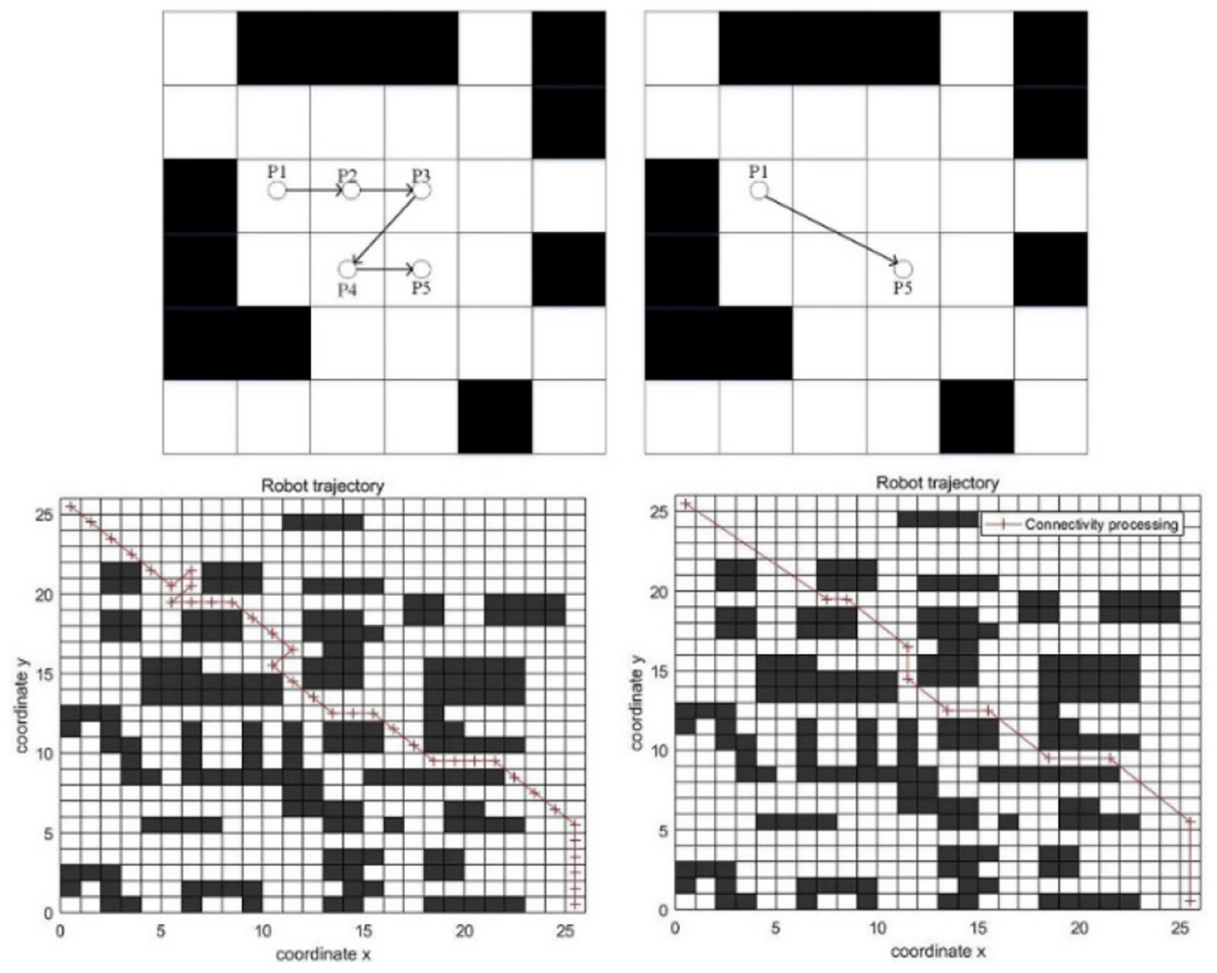
Connectivity processing.

Aiming at the path feature of the ant colony algorithm, which is composed of a series of nodes, the connectivity principle is proposed. Due to the limitation of step size, there are many redundant nodes. Connectivity processing is an effective method for eliminating redundant nodes, and its principle is shown in Figure 7.

As can be seen from the Figure 7, the line between P1 and P5 does not cross obstacles, so the two nodes are connected. P2, P3, and P4 are redundant nodes. After connectivity processing, the corner is reduced, and the path length is shortened, which is beneficial to the robot.

The connectivity processing of the complete path is shown in Figure 7.

As can be seen from the Figure 7, the left side is the path obtained by the classic ant colony algorithm. There are redundant nodes, and the path length is 43.6985. The right side is the path processed by connectivity. The corner is reduced, and the path length is 39.1901. It can be seen that the effect of connectivity processing is significant.

In addition to redundant nodes, the path smoothing also includes the smoothness operation of the corner. Therefore, this paper introduces the quadratic B-spline curve optimization method to optimize the corner.

In 1946, Schoenberg proposed a spline-based approach to approximate curves. In 1972, based on Schoenberg's work, Gordon and Riesenfeld proposed B-spline curves and a series of corresponding geometric algorithms. The B-spline curve is the generalization of the Bezier curve, which solves the problem that the Bezier curve is difficult to smooth transition at the endpoint. Besides, the B-spline curve has higher accuracy (Zeng et al., 2019). The definition of the B-spline curve is as follows.


(28) $$\begin{array}{rcl} P\left(t\right) & = & \overset{n}{\underset{i=0}{\sum }}P_{i}N_{i,k}\left(t\right) \end{array}$$

(29) $$\begin{array}{l} \begin{array}{ccc} N_{i,k}(t) & = & \frac{1}{k!}*\sum_{j=0}^{k-i}{\left(-1\right)}^{j}*C_{k+1}^{j}*{\left(t+k-i-j\right)}^{k} \end{array}\\ \;\begin{array}{c} 0\leq t\leq 1,i=0,1,\ldots ,k-1,C_{k+1}^{j} \end{array} \end{array}$$

(30) $$\begin{array}{ll} = & \frac{\left(n+1\right)!}{j!*\left(n+1-j\right)!} \end{array}$$

Where, *P*_*i*_is the original endpoint, *N*_*i, k*_(*t*)is the basic function, and*P*(*t*)is the set of points on the curve.

The quadratic B-spline method needs only three endpoints to construct a smooth curve. And it can meet the requirements of curve smoothness. Therefore, this paper selects the quadratic B-spline method to deal with the corner problem.

When*n* = 2in equation (28), the quadratic B-spline curve of the following form can be obtained through the spline basis function.


(31) $$\begin{array}{l} P(t)=\frac{1}{2}*{\left(1-t\right)}^{2}*P_{0}+\frac{1}{2}*(-2*t^{2}+2*t+1)\\ \;*P_{1}+\frac{1}{2}*t^{2}*P_{2} \end{array}$$

The quadratic B-spline method is shown below.

**Figure 8:**
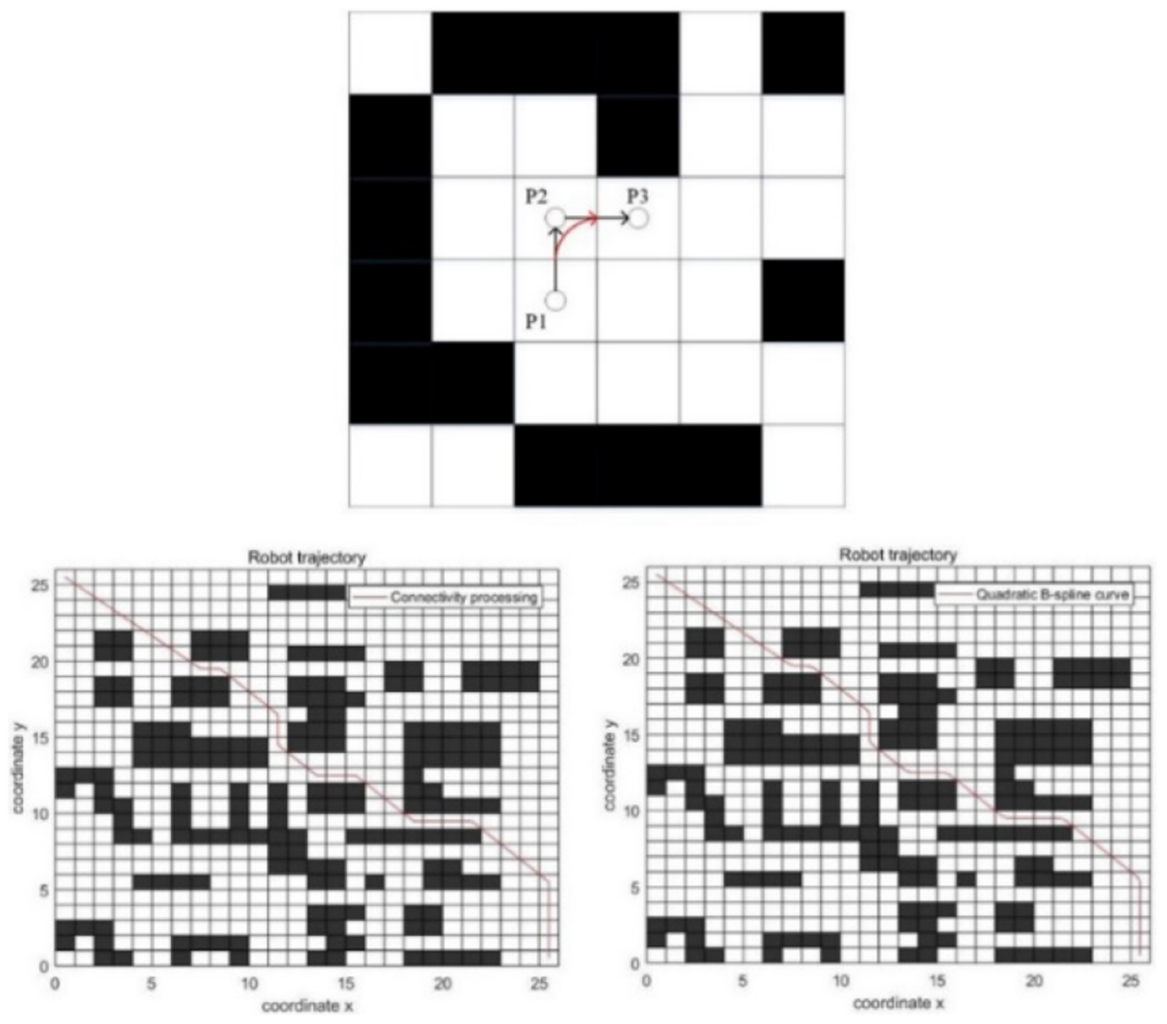
Quadratic B-spline curve.

As can be seen from the Figure 8, the black path is the original path, and the red path at the corner is the new path generated by the quadratic B-spline method.

The comparison results between the path after connectivity processing and the path generated by the quadratic B-spline method based on connectivity processing is shown in Figure 8.

As can be seen from the Figure 8, the corner processed by the quadratic B-spline method is smoother. The path length before processing is 39.1901, and the path length after processing is 38.9281.

There is almost no redundant node of the path processed by connectivity and the quadratic B-spline method, which is shorter than the path generated by the classic ant colony algorithm and is more suitable for mobile robots.

### Algorithm flow

**Figure 9:**
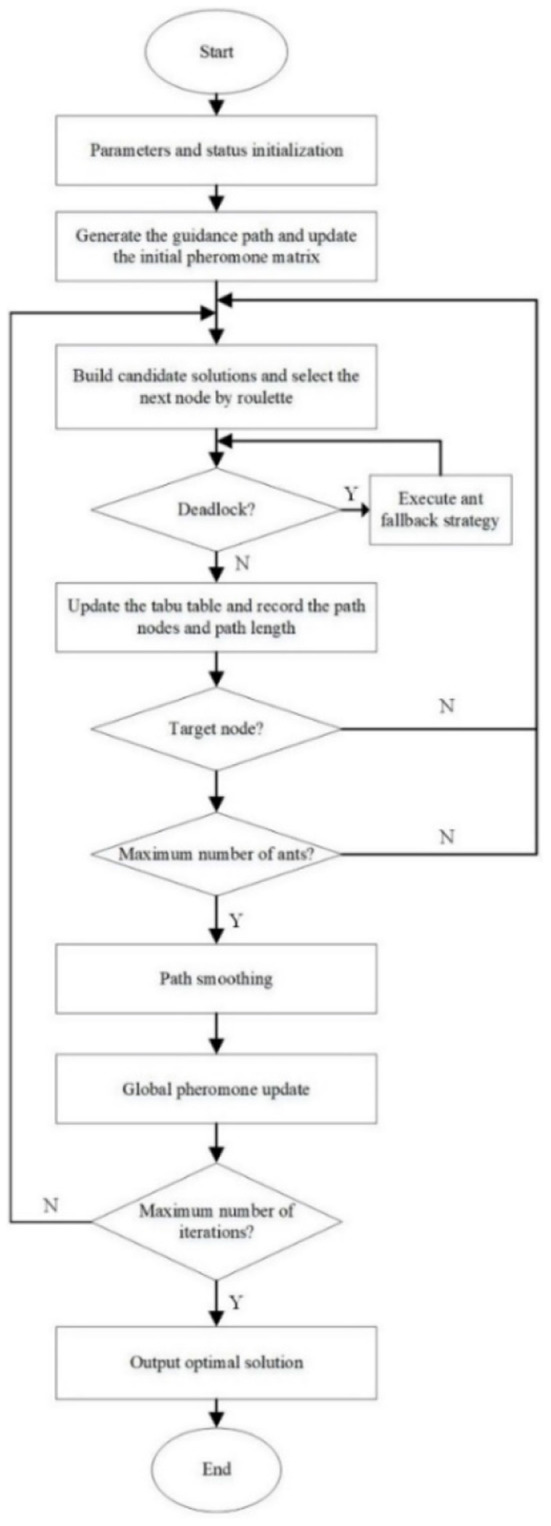
Flow chart of improved ant colony optimization algorithm.

To sum up, the flow of the improved ant colony algorithm is shown in Figure 9.

The execution steps of the algorithm are as follows.

Step 1: Initialize the parameters of the improved ant colony algorithm and Floyd algorithm.
Step 2: Set up the grid map, initialize the pheromone matrix and tabu list according to the guidance path generated by the Floyd algorithm.
Step 3: Build candidate solutions according to the tabu list and state transition rules and select the next node by roulette principle.
Step 4: Determine whether the ants fall into the deadlock. If so, execute the fallback strategy until the ants get out of the trap. Otherwise, continue step 5.
Step 5: Update the tabu list and record the path nodes and length.
Step 6: Determine whether the ant reaches the target node. If so, continue step 7. Otherwise, return to step 3.
Step 7: Determine whether the number of ants reaches the upper limit. If so, continue step 8. Otherwise, return to step 3.
Step 8: Smooth the path and update the global pheromone.
Step 9: Determine whether the maximum number of iterations is reached. If so, outputs the optimal solution and ends. Otherwise, return to step 3.
Step 10: Draw the algorithm iteration diagram and the optimal path curve.

## Experimental results and discussions

In this section, the effectiveness of the improved algorithm in path planning is verified through different scenarios. All experiments were performed using the same PC. The MATLAB (R2016b) programming platform was used to encode and implement all algorithms. In order to obtain real experimental results and avoid accidental situations, all experiments were carried out independently under the same experimental conditions.

The 26 × 26 scale grid map is adopted in this paper. There are three different environments, namely, the concentrated obstacle environment, the partially dispersed obstacle environment, and the decentralized obstacle environment. The algorithm in this paper, the classic ant colony algorithm, and the algorithm of Li et al. (2021), Luo et al. (2020), and Akka and Khaber (2018) are compared experimentally. The algorithm parameters are set as shown in Table 2.

**Table 2:** Parameter setting.

**Parameter**	
Starting point *S*	1
Target point *E*	676
Maximum number of iterations *K*	100
The number of ants *M*	50
Pheromone heuristic factor α	1
Expected heuristic factor β	6
Pheromone volatilization factor ρ	0.6
Pheromone intensity factor *Q*	1
Pheromone penalty evaporation coefficient λ	15

### Concentrated obstacle environment

**Figure 10:**
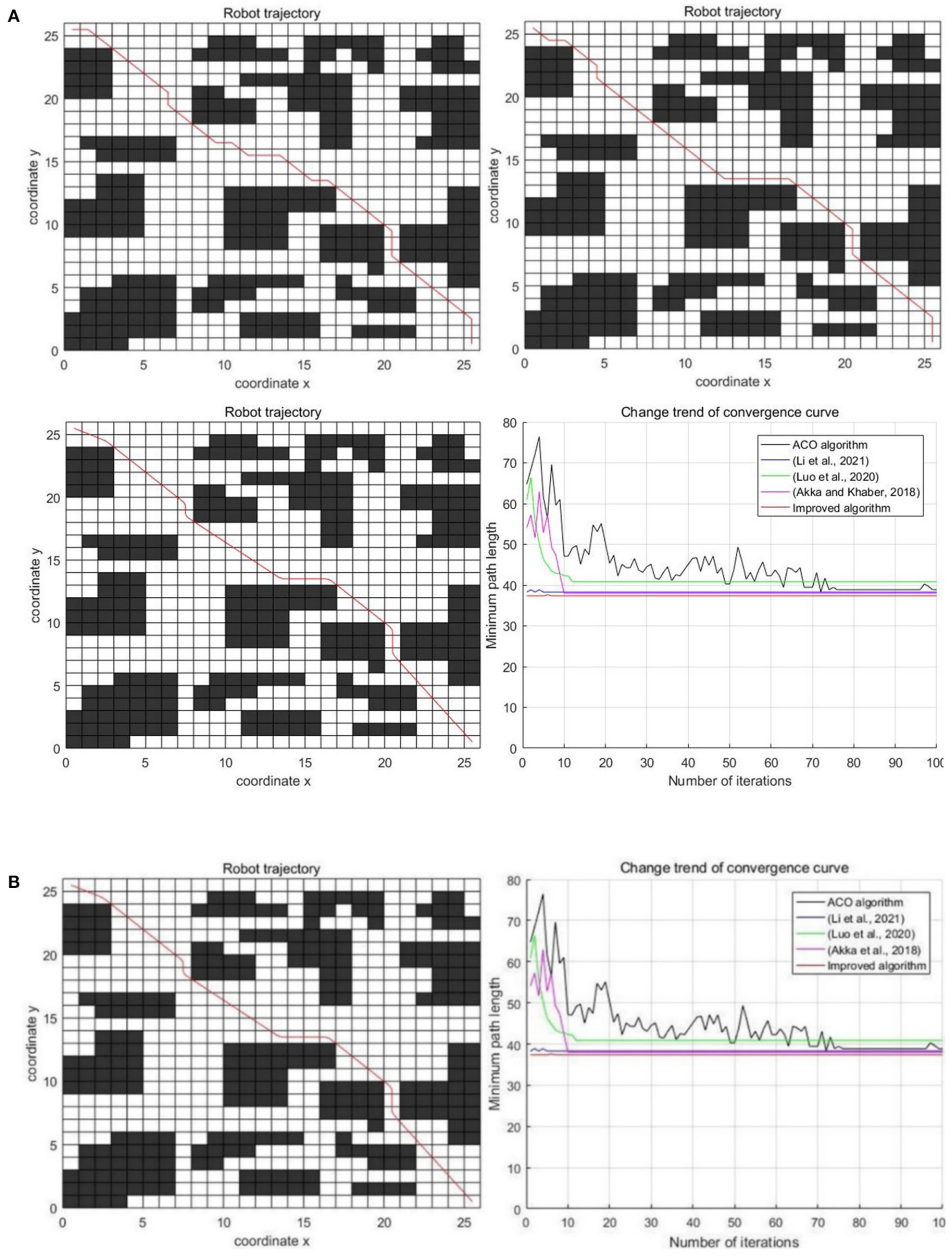
Experimental results of three algorithms in the concentrated obstacle environment. **(A)** Classic ant colony algorithm, Luo et al. (2020); Li et al. (2021), and Akka and Khaber (2018) **(B)** Improved algorithm and comparison of five algorithms.

In the concentrated obstacle environment with the 26 × 26 scale grid, the experimental results of five algorithms are shown in Figure 10.

The specific results of the experiment are shown in Table 3. Index 1 is the average path length, index 2 is the optimal path length, index 3 is the average number of iterations, and index 4 is the average number of corners.

**Table 3:** Comparison of five algorithms.

	**Index**	**Concentrated obstacle**	**Partially decentralized**	**Decentralized obstacle**
		**environment**	**obstacle environment**	**environment**
Classic ACO	1	39.2843	41.4578	43.2763
	2	38.8701	40.0416	41.4558
	3	70	65	75
	4	12	14	18
Li et al. (2021)	1	38.5772	39.9771	48.6639
	2	38.2843	39.1127	46.2543
	3	5	10	18
	4	9	12	15
Luo et al. (2020)	1	41.5772	40.4056	43.2132
	2	40.4807	39.6985	41.7990
	3	12	9	8
	4	15	15	16
Akka and Khaber (2018)	1	38.3045	40.8078	41.3356
	2	37.9793	39.6853	40.9214
	3	10	10	12
	4	10	13	16
Improved algorithm	1	37.5438	39.0204	39.6872
	2	37.2033	38.9281	39.1280
	3	7	11	10
	4	7	9	11

It can be seen from Figure 10 and Table 3 that the comprehensive performance of the improved algorithm in this paper is the best in the concentrated obstacle environment. In terms of the optimal path length, the improved algorithm is 4.29% less than the classic ant colony algorithm, 2.82% less than the algorithm in Li et al. (2021), 8.10% less than the algorithm in Luo et al. (2020), and 2.04% less than the algorithm in Akka and Khaber (2018). In terms of the average path length, the improved algorithm is 4.43, 2.68, 9.70, and 1.99% less than other algorithms, respectively. In terms of the average number of iterations, the improved algorithm is 63 times less, 2 times more, 5 times less and 3 times less than other algorithms, respectively. In terms of the average number of corners, the improved algorithm is 41.67, 22.22, 53.33, and 30% less than other algorithms respectively. To sum up, in the concentrated obstacle environment, the performance of the improved algorithm in this paper is better than the other four algorithms, including the classic algorithm.

### Partially decentralized obstacle environment

**Figure 11:**
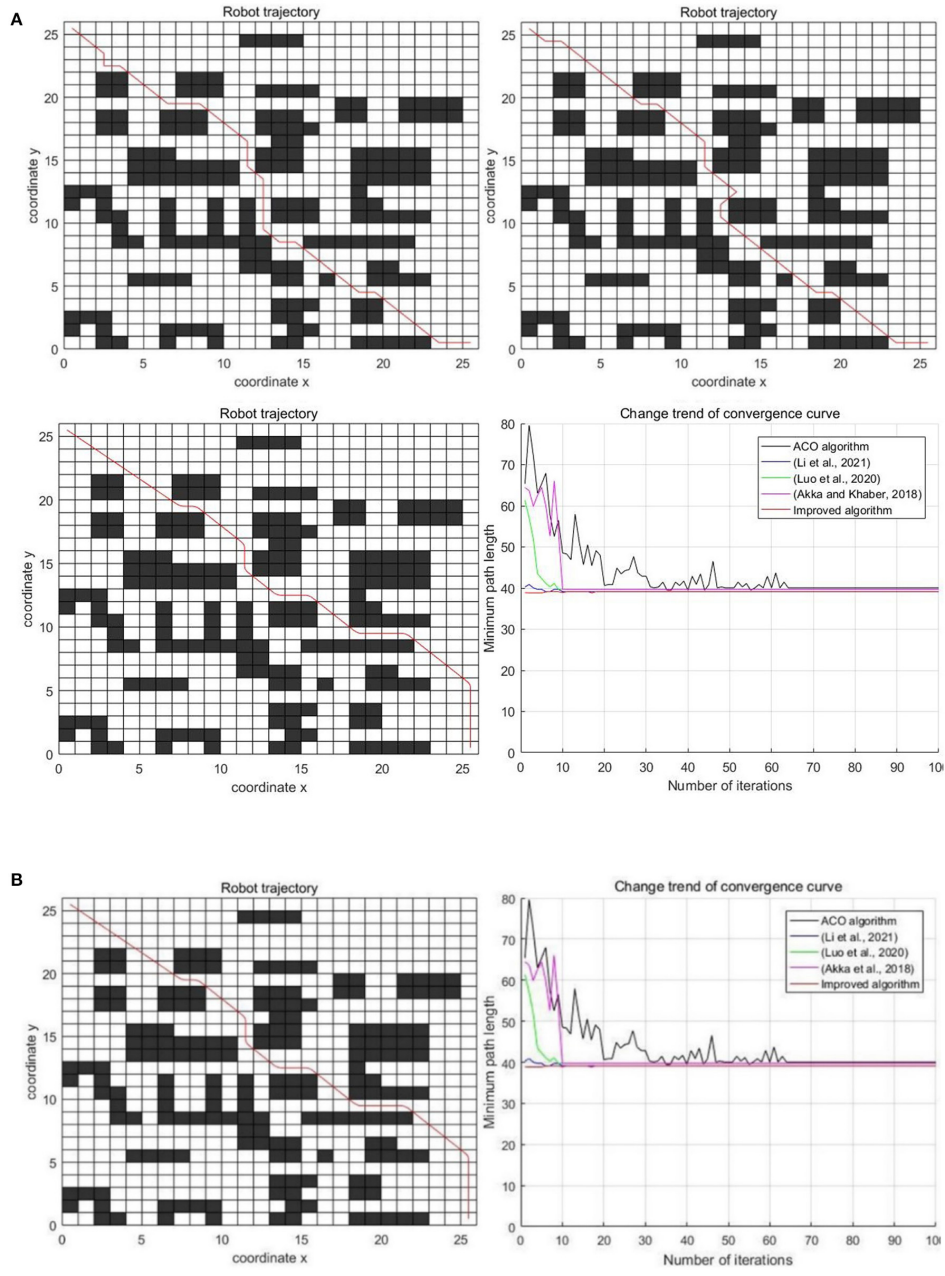
Experimental results of three algorithms in the partially decentralized obstacle environment. **(A)** Classic ant colony algorithm, Luo et al. (2020); Li et al. (2021), and Akka and Khaber (2018). **(B)** Improved algorithm and comparison of five algorithms.

In the partially decentralized obstacle environment with the 26 × 26 scale grid, the experimental results of five algorithms are shown in Figure 11.

The specific results of the experiment are shown in Table 3.

As can be seen from Figure 11 and Table 3, the performance of the improved algorithm in this paper is still better than that of other algorithms in the partially decentralized obstacle environment. In terms of the optimal path length, the improved algorithm is 2.87% less than the classic ant colony algorithm, 0.47% less than the algorithm in Li et al. (2021), 1.94% less than the algorithm in Luo et al. (2020), and 1.91% less than the algorithm in Akka and Khaber (2018). In terms of the average path length, the improved algorithm is 5.88, 2.39, 3.43, and 4.38% less than other algorithms respectively. In terms of the average number of iterations, the improved algorithm is 54 times less, 1 time more, 2 times more and 1 time more than other algorithms respectively. In terms of the average number of corners, the improved algorithm is 35.71, 25, 40, and 30.77% less than other algorithms respectively. It can be seen from the above that the performance of the improved algorithm in this paper still has certain advantages in the partially decentralized obstacle environment.

### Decentralized obstacle environment

**Figure 12:**
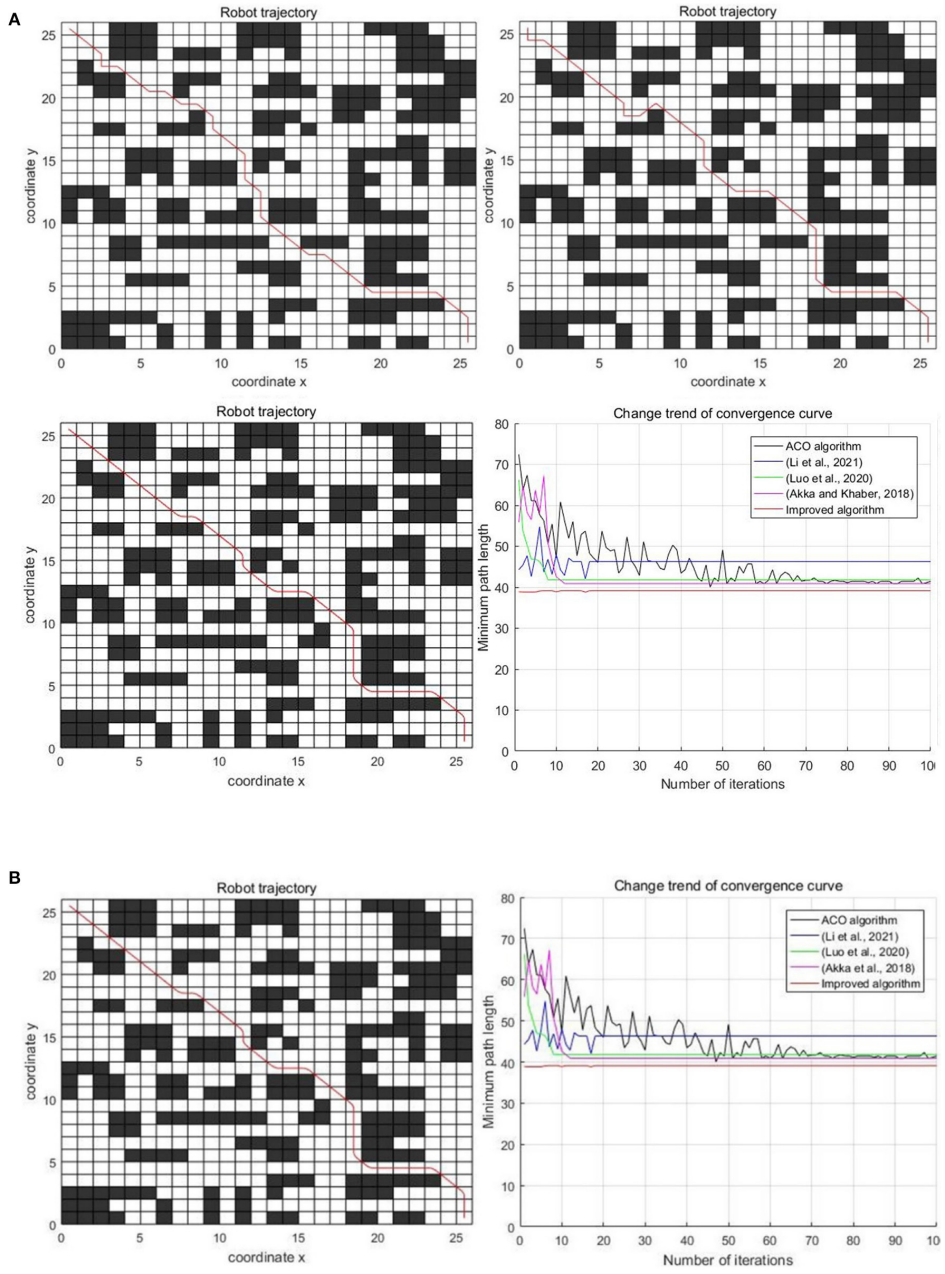
Experimental results of three algorithms in the decentralized obstacle environment. **(A)** Classic ant colony algorithm, Luo et al. (2020); Li et al. (2021), and Akka and Khaber (2018). **(B)** Improved algorithm and comparison of five algorithms.

In the decentralized obstacle environment with the 26×26 scale grid, the experimental results of five algorithms are shown in Figure 12. The specific results of the experiment are shown in Table 3.

It can be seen from Figure 12 and Table 3 that the improved algorithm in this paper has more obvious advantages than other algorithms in the decentralized obstacle environment. In terms of the optimal path length, the improved algorithm is 5.62% less than the classic ant colony algorithm, 15.41% less than the algorithm in Li et al. (2021), 6.39% less than the algorithm in Luo et al. (2020), and 4.38% less than the algorithm in Akka and Khaber (2018). In terms of the average path length, the improved algorithm is 8.29, 18.45, 8.16, and 3.99% less than other algorithms, respectively. In terms of the average number of iterations, the improved algorithm is 65 times less, 8 times less, 2 times more and 2 times less than other algorithms, respectively. In terms of the average number of corners, the improved algorithm is 38.89, 26.67, 31.25, and 31.25% less than other algorithms, respectively. From the above comparisons, as the complexity of the environment increases, the improved algorithm in this paper always has significant advantages.

From the above experiments, it can be seen that in the simple environment, except for the classic ant colony algorithm, the other three algorithms are close to the improved algorithm. As the complexity of the environment increases, the indicators of the five algorithms have changed, and the performance of the improved algorithm has always remained stable, which has been better than the other four algorithms, including the classic algorithm. Among the five algorithms, the improved algorithm is the best, which is most conducive to the energy-saving and stable operation of the robot.

## Conclusion

The ant colony algorithm is widely used in robot path planning. However, the classic ant colony algorithm still has the problems of slow convergence speed and easily fall into the local optimal solution. Therefore, this paper proposes an improved ant colony algorithm. Firstly, the Floyd algorithm is introduced to generate the guidance path to optimize the initial pheromone matrix and effectively accelerate the initial convergence speed of the ant colony algorithm. Ant fallback strategy can help avoid ants dying due to the deadlock dilemma and improve the global search ability of the algorithm. The improved heuristic function proposed by referring to the gravity concept in the APF method accelerates the convergence speed of the ant colony algorithm. It makes up for the influence of the fallback strategy on the convergence rate. The pheromone updating method based on a multi-objective optimization idea and dynamic principle considers the path length, path security, and path energy consumption. It helps the ant colony algorithm avoid the local optimal solution and improves the comprehensive performance of the algorithm, which is more suitable for mobile robots. Connectivity processing and the quadratic B-spline method effectively reduce the redundant nodes of the path, improve the smoothness of the path and further shorten the path length.

Through experimental comparisons, as can be seen, the improved algorithm has strong stability. From the simple obstacle environment to the complex obstacle environment, it can always maintain the optimal comprehensive performance, the shortest path, and the least corner. The problems of the classic ant colony algorithm has been solved. In addition, the multi-objective optimization idea and the node optimization method introduced in the improved algorithm can effectively help the mobile robot to save energy and improve the work efficiency.

## Data availability statement

Publicly available datasets were analyzed in this study. This data can be found here: https://pan.baidu.com/s/1QQiLWLKRe3_SM1QdnwwaqA?pwd=100d.

## Author contributions

LW and HW proposed this contribution, verified, and concluded simulation results. XC and BW gave suggestions for manuscript writing. All authors contributed to the article and approved the submitted version.

## Funding

This work was supported by the National Natural Science Foundation of China under Grant (31901400 and 61903351), Natural Science Foundation of Zhejiang under Grant LY22F030009, Special Project for Cultivating Young Scientific and Technological Talents (Class A) (2022YW20), National Key Technologies Research and Development of China (2018YFB2101004).

## Conflict of interest

The authors declare that the research was conducted in the absence of any commercial or financial relationships that could be construed as a potential conflict of interest.

## Publisher's note

All claims expressed in this article are solely those of the authors and do not necessarily represent those of their affiliated organizations, or those of the publisher, the editors and the reviewers. Any product that may be evaluated in this article, or claim that may be made by its manufacturer, is not guaranteed or endorsed by the publisher.

## References

[B1] Akka K. Khaber F. (2018). Mobile robot path planning using an improved ant colony optimization. Int. J. Adv. Robot. Syst. 15:1729881418774673. 10.1177/172988141877467333378876

[B2] Ali H. Gong D. W. Wang M. Dai X. L. (2020). Path planning of mobile robot with improved ant colony algorithm and MDP to produce smooth trajectory in grid-based environment. Front. Neurorobotics 14:44. 10.3389/fnbot.2020.00044 32733227 PMC7363842

[B3] Chen H. Niu L. Ji Y. (2020). Research on path planning of robot based on adaptive ACS fused with SHAA neural network. Meas. Control 53, 1911–1919. 10.1177/0020294020959751

[B4] Chen X. Gao P. (2020). Path planning and control of soccer robot based on genetic algorithm. J. Ambient Intell. Humaniz. Comput. 11, 6177–6186. 10.1007/s12652-019-01635-1

[B5] Chen Y. Bai G. Zhan Y. Hu X. Liu J. (2021). Path planning and obstacle avoiding of the USV based on improved ACO-APF hybrid algorithm with adaptive early-warning. IEEE Access 9, 40728–40742. 10.1109/ACCESS.2021.3062375

[B6] Dai W. Wang L. Wang B. Cui X. Li X. (2022). Research on WNN greenhouse temperature prediction method based on GA. Phyton Int. J. Exp. Bot. 91, 2283–2296. 10.32604/phyton.2022.021096

[B7] Dai X. Long S. Zhang Z. Gong D. (2019). Mobile robot path planning based on ant colony algorithm with A^*^ heuristic method. Front. Neurorobotics 13:15. 10.3389/fnbot.2019.00015 31057388 PMC6477093

[B8] Guo X. Ji M. Zhao Z. Wen D. Zhang W. (2020). Global path planning and multi-objective path control for crewless surface vehicle based on modified particle swarm optimization (PSO) algorithm. Ocean Eng. 216, 107693–107708. 10.1016/j.oceaneng.2020.107693

[B9] Hao Z. He S. (2008). Some discussions on the Floyd algorithm. J. Chongqing Univ. Technol. 22, 156–159. 10.3969/j.issn.1674-8425-B.2008.05.03934550899

[B10] Khaksar W. Hong T. Khaksar M. Motlagh O. (2012). Sampling-based tabu search approach for online path planning. Adv. Robot. 26, 1013–1034. 10.1163/156855312X632166

[B11] Li E. Wang Y. (2022). Research on obstacle avoidance trajectory of mobile robot based on improved artificial potential field. Comput. Eng. Appl. 58, 296–304. 10.3778/j.issn.1002-8331.2108-0122

[B12] Li K. Liu S. Hu Q. Tang Y. (2021). Path planning for improved ant colony optimization algorithm based on corner constraints. J. Comput. Appl. 41, 2560–2568. 10.11772/j.issn.1001-9081.2020111713

[B13] Li X. Wang L. (2020). Application of improved ant colony optimization in mobile robot trajectory planning. Math. Biosci. Eng. 17, 6756–6774. 10.3934/mbe.2020352 33378876

[B14] Luo Q. Wang H. Zheng Y. He J. (2020). Research on path planning of mobile robot based on improved ant colony algorithm. Neural Comput. Appl. 32, 1555–1566. 10.1007/s00521-019-04172-234198374

[B15] Lyu D. Chen Z. Cai Z. Piao S. (2021). Robot path planning by leveraging the graph-encoded Floyd algorithm. Futur. Gener. Comp. Syst. 122, 204–208. 10.1016/j.future.2021.03.007

[B16] Mac T. Copot C. Tran D. DeKeyser R. (2016). Heuristic approaches in robot path planning: a survey. Robot. Auton. Syst. 86, 13–28. 10.1016/j.robot.2016.08.001

[B17] Ouyang X. Yang S. (2014). Obstacle avoidance path planning of mobile robot based on potential field grid method. Control Eng. China 21, 134–137. 10.3969/j.issn.1671-7848.2014.01.031

[B18] Pan Z. Li D. Yang K. Deng H. (2019). Multi-robot obstacle avoidance based on the improved artificial potential field and PID adaptive tracking control algorithm. Robotica 37, 1883–1903. 10.1017/S026357471900033X

[B19] Shi W. Wang K. (2009). Floyd algorithm for the shortest path planning of mobile robot. Chin. J. Sci. Instrum. 30, 2088–2092. 10.19650/j.cnki.cjsi.2009.10.014

[B20] Tao Y. Gao H. Ren F. Chen C. Wang T. Xiong H.. (2021). A mobile service robot global path planning method based on ant colony optimization and fuzzy control. Appl. Sci. 11, 3605–3619. 10.3390/app11083605

[B21] Tian Z. (2020a). Preliminary research of chaotic characteristics and prediction of short-term wind speed time series. Int. J. Bifurcation Chaos 30:2050176. 10.1142/S021812742050176X

[B22] Tian Z. (2020b). Short-term wind speed prediction based on LMD and improved FA optimized combined kernel function LSSVM. Eng. Appl. Artif. Intell. 91:103573. 10.1016/j.engappai.2020.103573

[B23] Tian Z. (2021a). Approach for short-term traffic flow prediction based on empirical mode decomposition and combination model fusion. IEEE Trans. Intell. Transp. Syst. 22, 5566–5576. 10.1109/TITS.2020.2987909

[B24] Tian Z. (2021b). Modes decomposition forecasting approach for ultra-short-term wind speed. Appl. Soft. Comput. 105:107303. 10.1016/j.asoc.2021.107303

[B25] Tian Z. Chen H. (2021a). A novel decomposition-ensemble prediction model for ultra-short-term wind speed. Energy Conv. Manag. 248:114775. 10.1016/j.enconman.2021.114775

[B26] Tian Z. Chen H. (2021b). Multi-step short-term wind speed prediction based on integrated multi-model fusion. Appl. Energy 298:117248. 10.1016/j.apenergy.2021.117248

[B27] Tian Z. Li H. Li F. (2021). A combination forecasting model of wind speed based on decomposition. Energy Rep. 7, 1217–1233. 10.1016/j.egyr.2021.02.002

[B28] Tian Z. Li S. Wang Y. (2020). A prediction approach using ensemble empirical mode decomposition-permutation entropy and regularized extreme learning machine for short-term wind speed. Wind Energy 23, 177–206. 10.1002/we.2422

[B29] Wang L. (2020). Path planning for unmanned wheeled robot based on improved ant colony optimization. Meas. Control 53, 1014–1021. 10.1177/0020294020909129

[B30] Wang L. Liu L. Qi J. Peng W. (2020a). Improved quantum particle swarm optimization algorithm for offline path planning in AUVs. IEEE Access 8, 143397–143411. 10.1109/ACCESS.2020.3013953

[B31] Wang L. Wang B. (2020a). Construction of greenhouse environment temperature adaptive model based on parameter identification. Comput. Electron. Agric. 174:105477. 10.1016/j.compag.2020.105477

[B32] Wang L. Wang B. (2020b). Greenhouse microclimate environment adaptive control based on a wireless sensor network. Int. J. Agric. Biol. Eng. 13, 64–69. 10.25165/j.ijabe.20201303.5027

[B33] Wang L. Wang B. Zhu S. (2020b). Multi-model adaptive fuzzy control system based on switch mechanism in a greenhouse. Appl. Eng. Agric. 36, 549–556. 10.13031/aea.13837

[B34] Wang X. Shi H. Zhang C. (2020c). Path planning for intelligent parking system based on improved ant“‘ colony optimization. IEEE Access 8, 65267–65273. 10.1109/ACCESS.2020.2984802

[B35] Xiao S. Tan X. Wang J. (2021). A simulated annealing algorithm and grid map-based UAV coverage path planning method for 3D reconstruction. Electronics 10, 853–868. 10.3390/electronics10070853

[B36] Xiong N. Zhou X. Z. Yang X. Q. Xiang Y. Ma J. Y. (2021). Mobile robot path planning based on time taboo ant colony optimization in dynamic environment. Front. Neurorobotics 15:642733. 10.3389/fnbot.2021.642733 33732132 PMC7956960

[B37] Xiong X. Y. Min H. T. Yu Y. B. Wang P. Y. (2020). Application improvement of A^*^ algorithm in intelligent vehicle trajectory planning. Math. Biosci. Eng. 18:1. 10.3934/mbe.2021001 33525078

[B38] Yang H. Qi J. Miao Y. Sun H. Li J. (2019). A new robot navigation algorithm based on a double-layer ant algorithm and trajectory optimization. IEEE Trans. Ind. Electron. 66, 8557–8566. 10.1109/TIE.2018.2886798

[B39] Yang J. (2020). Application of Floyd algorithm in designing a coastal tourism route optimization system. J. Coast. Res. 106, 668–671. 10.2112/SI106-151.1

[B40] Yao Y. Liang X. Li M. Yu K. Chen Z. Ni C.. (2021). Path planning method based on D^*^ lite algorithm for unmanned surface vehicles in complex environments. China Ocean Eng. 35, 372–383. 10.1007/s13344-021-0034-z

[B41] Yi G. Feng Z. Mei T. Li P. Jin W. Chen S. (2019). Multi-AGVs path planning based on improved ant colony algorithm. J. Supercomput. 75, 5898–5913. 10.1007/s11227-019-02884-9

[B42] Zeng D. Zhu P. Zhang P. Chen H. (2019). Cubic B-spline curve for obstacle avoidance trajectory planning of unmanned vehicle. J. Tongji Univ. 47, 159–163.

[B43] Zhang C. Pu J. Si Y. (2021). An adaptive improved ant colony system based on population information entropy for path planning of mobile robot. IEEE Access 9, 24933–24945. 10.1109/ACCESS.2021.3056651

[B44] Zhang C. Pu J. Si Y. Sun L. (2020). Survey on application of ant colony algorithm in path planning of mobile robot. Comput. Eng. Appl. 56, 10–19. 10.3778/j.issn.1002-8331.1912-0160

[B45] Zhang W. Ma Y. Zhao H. Zhang L. Li Y. Li X. (2019). Obstacle avoidance path planning of intelligent mobile based on improved fireworks-ant colony hybrid algorithm. Control Decis. 34, 335–343. 10.13195/j.kzyjc.2017.087034630554

[B46] Zheng Y. Luo Q. Wang H. Wang C. Chen X. (2020). Path planning of mobile robot based on adaptive ant colony algorithm. J. Intell. Fuzzy Syst. 39, 5329–5338. 10.3233/JIFS-189018

